# Avoidant/Restrictive Food Intake Disorder and Food Neophobia in Adult Patients with Food Allergy: A Preliminary Study

**DOI:** 10.3390/nu18060913

**Published:** 2026-03-13

**Authors:** Laura Polloni, Lucia Ronconi, Valentina De Fazio, Roberta Bonaguro, Francesca Lazzarotto, Alice Toniolo, Rossana Schiavo, Antonella Muraro

**Affiliations:** 1 Food Allergy Referral Centre, Veneto Region, Department of Women’s and Children’s Health, Padua University Hospital, 35128 Padua, Italy; 2 Unit of Psychology, Padua University Hospital, 35128 Padua, Italy; 3 Computer and Statistical Services, Multifunctional Pole of Psychology, University of Padua, 35100 Padua, Italy

**Keywords:** picky eating, eating behaviour, ARFID, food neophobia, food allergy, anaphylaxis, mental health, anxiety, elimination diet

## Abstract

**Background/Objectives**: Patients with food allergy (FA) may exhibit dysfunctional eating behaviours and/or food aversions that extend beyond the necessary allergen elimination diet and may result in avoidant/restrictive food intake disorder (ARFID) or food neophobia (FN); however, no data are available on adults. This study aimed to explore ARFID, FN, FA anxiety, and eating styles in adults with FA, analysing influences of sociodemographic and clinical factors. **Methods**: This cross-sectional preliminary study involved 79 adults with FA, who completed the Nine Item ARFID screen (NIAS), Food Neophobia Scale (FNS), Scale of Food Allergy Anxiety (SOFAA), and Dutch Eating Behaviour Questionnaire (DEBQ—brief). Pearson and Spearman correlation coefficients and multiple linear regressions were performed (*p* < 0.05). **Results**: In total, 25% and 73% of participants scored positively for ARFID and FN, respectively. A positive correlation was observed between FN and ARFID levels (*p* < 0.006), and between FN and FA anxiety (*p* < 0.001). Current psychological problems positively correlated with ARFID (*p* = 0.004), FN (*p* = 0.006), and FA anxiety scores (*p* = 0.03). Restrained eating was positively associated with female gender (*p* < 0.001), and ARFID (*p* = 0.002) and FN scores (*p* = 0.028). External eating was negatively correlated with ARFID (*p* = 0.004). Adrenaline auto-injector (AAI) prescription was negatively associated with ARFID (*p* < 001) and restrained eating (*p* = 0.006), while previous anaphylaxis was negatively associated with ARFID (*p* = 0.020) and positively associated with external eating (*p* = 0.021). Multiple logistic regression models confirmed that restrained eating was associated with a higher probability of both ARFID (*p* = 0.031) and FN (*p* = 0.074). **Conclusions**: Clinicians should be aware of the risk of ARFID and FN among adult patients with FA and recommend appropriate psychological and dietary support. Further studies are needed to better understand the protective and precipitating factors of ARFID and FN to develop effective prevention and treatment strategies.

## 1. Introduction

### 1.1. Food Allergy and Eating Difficulties

Food allergy (FA) is an adverse reaction to food that is mediated by the immune system. It affects approximately 3–10% of children and up to 10% of adults [1]. A strictly allergen-free diet and an emergency protocol that may include self-injectable adrenaline in the event of accidental exposure remain the most effective treatment options. Oral immunotherapy (OIT) is a promising treatment; however, it is a challenging process. It is prolonged, it requires allergen daily consumption, and, moreover, adverse reactions—ranging from mild reactions to anaphylaxis—are often experienced during treatment [2,3]. Therefore, patients with severe FA, and especially those allergic to multiple foods, frequently remain on an elimination diet. FA is associated with an increased risk of nutritional deficiencies, impaired growth, and mental health burden [4,5]. In some cases, patients may exhibit dysfunctional eating behaviours and/or food aversions that go beyond a medically necessary allergen elimination diet [6,7]. Indeed, chronic diseases involving strict dietary adherence have been associated with an increased risk of eating disorders. A recent systematic review has shown that in samples with IgE-mediated FA, the prevalence of anorexia nervosa and/or bulimia nervosa ranged from 17.6 to 61%, while avoidant/restrictive food intake disorder (ARFID) had a prevalence of 62.9% among children with FA [8].

### 1.2. ARFID

ARFID is a feeding and eating disorder introduced in the DSM-5 [9]. It is characterized by avoidant or restrictive eating patterns that are not determined by fear of weight gain or body image disturbances. Rather, avoidance or restriction is driven by factors such as lack of interest in food or eating, sensory sensitivity (e.g., to textures, odours, appearance of food), or fear of potential consequences (e.g., vomiting or choking). ARFID may manifest as significant restriction in the quantity or variety of food intake that may lead to weight loss, growth retardation, nutritional deficiencies, dependence on tube feedings or supplements, and impaired psychosocial functioning [9,10]. It comprises age-inappropriate feeding behaviours, including food neophobia (FN) [11,12].

Other characteristic behaviours include eating smaller food portions, early signalling of satiety, lack of appetite, and anxiety during eating. ARFID is supposed to have a multifactorial aetiology: biological, medical, psychological, and environmental factors, and their interaction, are likely to contribute to the pathogenesis [13]. Negative experiences with food, including allergic reactions, may contribute to the onset of ARFID [13,14,15,16]. In these cases, food restriction would aim to avoid the discomfort associated with the condition or the aversion to food resulting from treatment, but it becomes a source of nutritional risk and psychosocial distress.

### 1.3. FN

FN is defined as refusal or reluctance to eat new or unfamiliar foods [11,12]. It can significantly influence food choices and diet quality. FN is considered a natural developmental stage in children between the ages of 2 and 6 and is evolutionary in nature because it protects individuals from ingesting potentially dangerous substances. However, if FN persists and increases, it becomes a disruptive behaviour that can lead to severe nutritional deficiencies and psychosocial impairments. FN can be inherited or displaced by environmental influences [11]. Patients with FA may develop fear related to eating due to the risk of allergic reactions, which can exacerbate avoidance behaviours and diffidence towards new food [14]. A higher risk of FN is strongly correlated with a higher risk of ARFID [13,14].

### 1.4. FA Anxiety

The relationship between FA and anxiety is attributed to food allergy-specific fears and anxieties, rather than a general propensity for anxiety. Some anxiety, short-lived and related to key events, is predictable and may even be beneficial if it promotes allergen avoidance and emergency preparedness. However, increased anxiety is linked to maladaptive coping [14,17]. Food allergy anxiety is excessive anxiety about the potential for accidental and fatal cross-contamination and the fear of experiencing an allergic reaction [18]. It can lead to excessive/unnecessary restrictions that are disproportionate to the individual’s level of risk, significantly contributing to burden, reduced quality of life, and poorer management.

### 1.5. Aim of the Present Study

Research on ARFID and FN has focused primarily on children and adolescents, and very few data are available on adults [13,15,16]. Białek-Dratwa et al. [13] found that 15.2% of adults reported food selectivity and a lack of interest in food, and 11.0% showed fear and apprehension toward food. Although a high prevalence of eating/feeding dysfunctions and an increased risk of poor nutrition and negative adjustment outcomes have been observed among paediatric patients with FA [7,8,14,19,20,21], no data are available on adults. Furthermore, the role played by sociodemographic, psychological, and dietary factors in the development of ARFID and FN is not yet clearly understood. This article, therefore, aimed to preliminarily investigate ARFID, FN, FA anxiety, and eating styles in a sample of adults with IgE-mediated FA, analysing the possible influence of sociodemographic (e.g., age, gender, education) and clinical factors (e.g., number of allergens, age at diagnosis, AAI prescription, asthma, psychological issues, previous anaphylaxis, OIT) as well as the possible association between variables.

## 2. Materials and Methods

### 2.1. Study Design

The present study used a quantitative cross-sectional design, using validated measures assessing ARFID [12,22], FN [23,24], FA anxiety [18], and eating styles [25,26].

### 2.2. Participants and Procedure

The study involved 79 adult patients (aged >18 years) with a medical diagnosis of FA. Sociodemographic characteristics and clinical variables are reported in Table 1. Adult patients attending the Food Allergy Referral Centre of Veneto Region, Padua, Italy, were introduced to the study, receiving an explanation of its objectives and procedures, during routine visits or via email. Patients were given a QR code to access the questionnaires. If interested, they participated in the study voluntarily and completely anonymously, providing informed consent and fully completing the questionnaires. The study was conducted in accordance with the Declaration of Helsinki. The Ethics Committee of the Padua University Hospital approved the research protocol. Personal data were managed in full compliance with privacy regulations in accordance with local legislation (Legislative Decree No. 196/2003 and EU Regulation 2016/679).

### 2.3. Instruments

Patients completed the following questionnaires:

*Nine Item Avoidant/Restrictive Food Intake Disorder Screen (NIAS)*

The NIAS [12,22] is a self-administered screening tool, consisting of 9 items, designed to evaluate patterns of avoidant or restrictive eating. It includes three subscales:

- Picky Eating: it assesses sensitivity or aversion to the sensory characteristics of food (e.g., “I dislike most foods that other people eat”);
- Appetite: it captures a general disinterest in eating or food (e.g., “I am not very interested in eating; I seem to have a smaller appetite than other people”);
- Fear: it measures fear of possible negative outcomes of eating (e.g., “I restrict myself to certain foods because I am afraid that other foods will cause gastrointestinal discomfort, choking, or vomiting”).

Individuals respond to each question on a scale from 0 (strongly disagree) to 5 (strongly agree). Subscales are each scored on a scale from 0 to 15, with higher scores indicating higher levels of each measure (picky eating, lack of interest, and fear). All items may also be summed to calculate a total score, ranging from 0 to 45, with higher scores indicating higher levels of avoidant/restrictive eating broadly. To screen for symptoms of ARFID, the cut-off scores (≥10 for picky eating and fear, ≥9 for appetite) suggested by Burton Murray et al. [12] were applied. The screening is considered positive if the individual has at least one subscale above the cut-off score. Cronbach’s alpha value showed good levels in the present research (alpha = 0.87 for NIAS total score, alpha = 0.82 for NIAS picky eating, alpha = 0.85 for NIAS appetite, and alpha = 0.83 for NIAS fear).

*Food Neophobia Scale (FNS)*

The FNS [23,24] is a 10-item scale which evaluates the reluctance toward or aversion to trying unfamiliar foods, often accompanied by fear or disgust, that can lead to the avoidance or rejection of the novel food. Each item is rated on a 7-point Likert scale, where 1 stands for “Strongly disagree”, while 7 stands for “Strongly agree”. All items can be summed to calculate a total score, ranging from 10 to 70, with higher scores indicating a higher level of aversion towards novel food. A score ≥36 is considered to indicate high risk for FN [24]. In the present research, a good Cronbach’s alpha was found (0.84).

*Scale of Food Allergy Anxiety (SOFAA)*

The SOFAA [18] (https://www.chop.edu/centers-programs/food-allergy-center/scales-food-allergy-anxiety-sofaa (accessed on 1 August 2025)) is a self-rated questionnaire to assess FA-related anxiety and related anxious avoidance behaviours over the past week. The adult version (SOFAA-A) is used to evaluate the corresponding adult perception of their FA-related anxiety and anxious avoidance. It consists of 21 items, with the responses scored on a 5-point rating scale (4 = almost always; 3 = often; 2 = sometimes; 1 = almost never; 0 = never). All items can be summed to calculate a total score, ranging from 0 to 84, with higher scores indicating greater anxiety. In the present study, a good Cronbach’s alpha was obtained (0.89).

*Dutch Eating Behaviour Questionnaire (DEBQ—brief)*

The DEBQ—brief [25,26] is a self-report questionnaire that describes three different eating styles:

- The emotional style refers to the habit of eating in response to negative emotion and distress;
- The restrained style reflects the tendency to reduce food intake to control bodyweight;
- The external eating style implies the tendency to eat in response to environmental food cues, such as sight or smell.

The questionnaire consists of 16 items in its brief version, rated on a scale ranging from 1 (=Never) to 5 (=Very often). Each item reflects the three eating styles: 6 items represent emotional eating (items 1-8-9-11-12-16); 5 items represent restrained eating (items 4-5-7-10-14); 5 items reflect external eating (items 2-3-6-13-15). Scores are obtained by dividing the sum of the item scores by the total number of items on that scale [26]. Cronbach’s alpha value showed good levels in the present study (emotional style alpha = 0.92; restrained style alpha = 0.82; and external style alpha = 0.78).

### 2.4. Statistical Analysis

First, descriptive statistics were calculated for the main variables of this study—the Scale of Food Allergy Anxiety (SOFAA), Nine Item Avoidant/Restrictive Food Intake disorder scale (NIAS), Food Neophobia Scale (FNS), and Dutch Eating Behaviour Questionnaire (DEBQ). For continuous variables the normality assumption was checked by skewness and kurtosis examination. Results for both indices (ranging from −0.74 to 1.28) showed no significant deviations from normality. Correlation between these variables was examined using the Pearson correlation coefficient. Next, the association of sociodemographic and clinical factors with these main variables was explored using the Pearson correlation coefficient for continuous (such as age) and binary variables (such as gender, education, AAI prescription, asthma, FA-related bullying, FA psychological support, current and previous psychological issues, previous anaphylaxis, hospital admission and AAI use), and using the Spearman correlation coefficient for ordinal variables (such as number of allergens, age at diagnosis, number of reactions and OIT). Finally, a multiple logistic regression model was used to evaluate the predictors of a positive screening for ARFID and FN symptoms.

## 3. Results

Sociodemographic characteristics and clinical variables of participants are shown in Table 1. The mean age was 32.92 (SD = 12.65; range, 18–67 years), 70% were female, and 43% had a high level of education. Most (69%) were multiallergic, with adrenaline auto-injector (AAI) prescription (82%), and had previous anaphylaxis (82%). About a third had a hospital admission, and 18% used an AAI. For the majority, the diagnosis of FA occurred in childhood (56%), and previous and current psychological issues were reported (67% and 57%, respectively). Approximately 20% had completed oral immunotherapy (OIT), and 25% were undergoing treatment. 

Regarding FA anxiety, as shown in Table 2, the average score of 21.62 is significantly below the midpoint of the scale (which ranges from 0 to 84). The same applies to the score recorded for ARFID, both for the total (11.57 on a scale from 0 to 45) and for the three subscales (4.61, 3.23, and 3.73, respectively, on a scale from 0 to 15). However, 25% showed a value above the cut-off in at least one subscale, thus indicating a positive screening for ARFID patterns. In contrast, the mean score exceeded the average value on the FNS (41.96 on a scale of 10 to 70), and 73% scored above the cut-off value.

A very strong positive correlation was observed between the NIAS subscales, and for this reason, we focused on the total NIAS score when examining correlations and in subsequent analyses (see Table 2). A strong positive correlation was observed between FN levels and ARFID levels (r = 0.49; *p* < 0.001), and a moderate positive correlation between FN and FA anxiety (r = 0.40; *p* < 0.001). Medium-strength correlations were also found between ARFID levels and eating styles, one positive with restrained eating (r = 0.34; *p* = 0.002) and one negative with external eating (r = −0.32; *p* = 0.004). Weak positive correlations were observed between FN levels and emotional eating (r = 0.25; *p* = 0.02) and restrained eating (r = 0.25; *p* = 0.02), and between FA anxiety levels and ARFID levels (r = 0.23; *p* = 0.04).

About the association of sociodemographic and clinical variables with the scales, as reported in Table 3, a medium-strength positive association was found between female gender and restrained eating style (r = 0.41; *p* < 0.001), as well as between FA-related bullying and asthma with FA anxiety (r = 0.37; *p* < 0.001 and r = 0.27; *p* = 0.01), and between psychological support for FA and current psychological problems with levels of ARFID (r = 0.27; *p* = 0.01 and r = 0.32; *p* = 0.004) and FN (r = 0.32; *p* = 0.004 and r = 0.31; *p* = 0.006), and, furthermore, between the number of reactions and levels of FN (r = 0.29; *p* = 0.01), and between previous anaphylaxis and external eating style (r = 0.26; *p* = 0.02). Several weakly positive correlations were also found between previous psychological problems with restrained eating style and FN (r = 0.25; *p* = 0.02 and r = 0.23; *p* = 0.04), and between psychological support for FA and current psychological problems with FA anxiety (r = 0.24; *p* = 0.03 and r = 0.24; *p* = 0.03). Conversely, a medium-strength negative association was observed between AAI prescription and levels of ARFID and restrained eating style (r = −0.38; *p* < 0.001 and r = −0.30; *p* = 0.006), and between previous anaphylaxis and ARFID levels (r = −0.26; *p* = 0.02). No significant correlations were found between hospitalisation, AAI use, and OIT (*p* > 0.05).

Finally, two multiple logistic regression models were tested for positive screening of ARFID and FN symptoms, which included FA anxiety and eating styles, controlling for all sociodemographic and clinical variables selected based on previous correlation analyses (see Table 4). Both models explained about one-third of the variance (33% and 34%, respectively). Restrained eating style was associated with a higher probability of both ARFID and FN (OR = 2.49; *p* = 0.03; OR = 2.02; *p* = 0.07). In addition, FA anxiety was also associated with a higher probability of FN (OR = 1.14; *p* = 0.002). Conversely, external eating style was associated with a lower likelihood of ARFID (OR = 0.24; *p* = 0.007), as was AAI prescription (OR = 0.05; *p* = 0.01). These results should be viewed with caution because some odds ratios, such as AAI prescription or previous anaphylaxis, showed very wide confidence intervals, implying greater variability in the data and higher uncertainty around the estimate.

## 4. Discussion

This is the first study, to our knowledge, to evaluate ARFID and FN in a group of adults with FA. Most participants were women, had suffered from multiple allergies since childhood, had a prescription for an AAI, had had previous anaphylaxis, and reported current or previous psychological difficulties such as anxiety, depression, and stress disorders. Approximately a quarter of the sample reported having experienced FA-related bullying, and the same percentage required psychological support due to FA.

### 4.1. Epidemiology of ARFID and FN in Adults with FA

The sample showed a relatively low mean score for ARFID and a high score for FN. Moreover, 25% of patients tested positive for ARFID screening and 73% for FN screening. The frequency of FN ranges from 13.4% to 26.4% [13,27,28,29,30] in European studies involving adults, while the frequency of ARFID is still not fully comprehended in the literature. A recent systematic review and meta-analysis [31] indicated an overall prevalence of ARFID of 4.51% or 11.14%, depending on the analytical model used. Prevalence ranged from 0.8% to 28% in non-clinical samples and from 0.8% to 64% in clinical samples [31]. Limited data [10] indicate that the prevalence of ARFID among adults can range from 0.3% in the general population [32] up to 11.0% in samples with eating disorders [33]. Data from a large online screening of eating disorders indicate that 4.7% of adult respondents tested positive for ARFID [34]. The enormous variability is due not only to the different characteristics of the populations and samples, but also to significant methodological heterogeneity between studies. In any case, our results showed a high prevalence of both ARFID and FN. Proctor et al. [35] recently highlighted that FA confers unique risk factors for ARFID, identifying three specific impact areas of FA that may determine the higher frequency of ARFID in this population. The unique characteristics of allergic reactions and medical treatment, the psychosocial impact of chronic vigilance and allergen avoidance, and the response to learning paradigms and physiological up-regulation may interfere with the development of adaptive feeding behaviour in both patients and caregivers [35].

### 4.2. ARFID, FN, and FA Anxiety

ARFID has been associated with multimorbidity, including mood disorders and somatic symptoms [31,36]. In line with previous studies, we found a strong positive correlation between ARFID and FN levels and a significant positive correlation between FA anxiety and FN and ARFID [10,13,15,31,33,36]. A reasonable anxiety—short-lived and related to specific events—is acceptable, and can also be adaptive when it supports effective FA management behaviours (e.g., reading labels, carrying drugs). However, excessive anxiety does not improve adherence. Rather, high levels of anxiety are counterproductive and related to maladaptive coping strategies, such as strict dietary restrictions beyond medical indications that can include avoidance of new and unfamiliar foods or preparations [15,17]. Individuals with FA are accustomed to paying particular attention to sensations in the oral and gastrointestinal areas, especially when tasting new foods, for fear of possible reactions. FA-related anxiety may amplify sensations or even be a barrier to tasting. In a previous study, we found that 11.1% of children never ate or rarely ate the tested food after a negative oral food challenge (OFC). Consumption of reintroduced food was positively correlated with the child’s interest in tasting new foods before and after OFC and maternal anxiety [37].

### 4.3. ARFID, FA, and Psychological Distress

In our sample, 57% and 67% of respondents, respectively, reported current or previous psychological problems (e.g., anxiety, depression, stress symptoms), and 23% reported having experienced bullying related to FA. However, only 25% reported having received a psychological assessment and support for their FA issues. The data are consistent with a global study reporting that 73% of patients/caregivers expressed FA-related distress, but less than 20% were assessed or screened [38]. We found that current psychological problems positively correlated with FA anxiety, ARFID, and FN scores, highlighting the association between psychological distress and eating difficulties in FA. Previous bullying episodes were confirmed to be related to FA-related anxiety [17,39], but no association with eating issues emerged. Gupta et al. [40] argued that patients with FA experience lower levels of “food freedom” than non-allergic individuals, which can lead to psychological, emotional, social, and nutritional difficulties. Furthermore, food avoidance patterns can affect neuroinflammatory states and the gut microbiome [40,41]; these changes may be associated with neuropsychiatric symptoms such as anxiety and depression. The authors concluded that targeted psychological counselling should be implemented [40].

### 4.4. ARFID, FN, and Eating Styles

Restrained eating habits were positively associated with female gender, previous psychological issues, and ARFID and FN scores. This finding confirms one of the central characteristics of both ARFID and FN, which is a quantitative/qualitative dietary restriction [10,11,12,13]. In patients with FA, this restriction goes beyond an allergen elimination diet and seems to be related more to an extreme and maladaptive attempt to protect oneself from food rather than to weight control. Emotional eating was weakly correlated only with FN and was the least reported eating style by participants. Emotional eating means eating to suppress or soothe negative emotions, such as stress, anger, boredom, and sadness [25,26]. Patients with FA may be unable to use food as a source of reassurance and comfort, as it is generally a focus of attention and, in many cases, worry. However, patients with FN may consume familiar foods as a source of comfort and for a sense of control. External eating, characterized by the tendency to eat in response to environmental food cues, such as sight or smell [25,26], was negatively correlated with ARFID. It is, indeed, usually associated with overeating rather than eating restriction [26].

### 4.5. ARFID, FN, and FA Clinical Variables

Prescribing AAIs was negatively associated with ARFID and restrained eating. It is plausible that providing this lifesaving drug makes adult patients feel safer, as already highlighted in the literature [42]. Previous anaphylaxis was found to be negatively associated with ARFID and positively associated with external eating. We hypothesize that patients with FA who are guided by environmental food cues may be less attentive or inclined to carry out the necessary checks and therefore more at risk of contamination or accidental ingestion. On the other hand, the excessive restrictions, lack of interest, and/or apprehension towards food that characterize ARFID could actually have a protective effect with respect to the possibility of incurring anaphylaxis, but to the detriment of the patient’s nutritional and psychological health. Finally, a greater number of reactions was correlated with a higher FN score. It is possible that repeated reactions may foster fixation on certain foods considered safe, decreasing openness to new foods or preparations. However, the correlational design does not allow us to draw causal conclusions; therefore, these hypotheses require further studies and confirmation. Other variables, such as hospital admission, AAI use, OIT, and number of allergens, did not show significant correlations with any variable in our sample.

## 5. Strengths and Limitations

In terms of strengths, this study examined ARFID and FN among adult patients with FA for the first time, providing initial data on the frequency of these eating disorders in the adult allergic population and offering a preliminary perspective on the influence of sociodemographic, clinical, psychological, and eating attitude factors. On the limitation side, a relatively small sample size may limit external validity and generalizability by failing to capture the complexity of the population. Our sample consists primarily of patients with severe FA treated at a tertiary care centre, and most participants were female. It could be that patients most in need of support or interested in the study topic were willing to participate. These aspects may limit the generalizability of the results to a broader population of adults with FA. The study used self-report questionnaires, which may be more prone to bias due to social desirability or inaccurate responses, although they have the advantage of capturing the patient’s perspective. Furthermore, the cross-sectional design does not allow for conclusions of causality. Finally, the results emerging from multiple logistic regression models must be interpreted with caution, because some odds ratios, such as AAI prescription or previous anaphylaxis, have very wide confidence intervals, which is a sign of instability in the estimates, probably a consequence of the small sample size. Further longitudinal studies on larger samples are needed to confirm these preliminary findings.

## 6. Conclusions

Finally, restrained eating style was confirmed to be associated with a higher probability of both ARFID and FN. FA anxiety was also associated with a higher probability of FN. Conversely, external eating style and AAI prescription were associated with a lower likelihood of ARFID, although these results should be considered with caution, due to the preliminary nature of the study. In conclusion, ARFID and FN appeared to be frequent, strongly correlated with each other, and associated with patients’ mental health, patients’ dietary approach, and some FA-related clinical variables, rather than with sociodemographic factors. In some cases, FA can be further complicated by extreme food aversion and self-imposed dietary restrictions that go beyond medically necessary dietary restrictions. These additional maladaptive eating behaviours add to the overall burden of FA, can cause nutritional deficiencies, and may contribute to a deterioration in patients’ quality of life [7]. ARFID and FN are complex disorders influenced by many factors, including sensory and psychological factors, genetics, and experience [13]. Understanding these factors is essential for developing effective prevention and treatment strategies for individuals suffering from these disorders. Patients with FA are an important target for intervention, given their vulnerability to psychological and nutritional issues. Clinicians should be aware of the risk of ARFID and FN among adult patients with FA and recommend appropriate psychological and dietary support. Further studies, particularly in the adult population, are needed.

## Figures and Tables

**Table 1:** Sociodemographic and clinical variables of adults with FA (n = 79).

Variables	Mean (SD); n (%)
Age (years)	32.92 (12.65)
Gender (female)	55 (70%)
Education (degree)	34 (43%)
Number of allergens	
1	24 (30%)
2	12 (15%)
3 or more	43 (54%)
Age at diagnosis	
Childhood	44 (56%)
Adolescence	12 (15%)
Adulthood	23 (29%)
AAI prescription	65 (82%)
Asthma	37 (47%)
FA-related bullying	18 (23%)
FA psychological support	20 (25%)
Current psychological issues	45 (57%)
Previous psychological issues	53 (67%)
Previous anaphylaxis	65 (82%)
Number of reactions	
0	14 (18%)
1	21 (27%)
2	19 (24%)
3 or more	25 (32%)
Hospital admission	29 (37%)
AAI use	14 (18%)
OIT	
No	44 (56%)
Yes, currently in progress	20 (25%)
Yes, already completed	15 (19%)

**Table 2:** Descriptive statistics of Scale of Food Allergy Anxiety (SOFAA), Nine Item Avoidant/Restrictive Food Intake disorder scale (NIAS), Food Neophobia Scale (FNS) and Dutch Eating Behaviour Questionnaire (DEBQ), and Pearson correlation coefficients between measures.

Measures		1	2	3	4	5	6	7	8	9
1.	SOFAA Total	-								
2.	NIAS Picky Eating	0.20	-							
3.	NIAS Appetite	0.12	0.41 ***	-						
4.	NIAS Fear	0.22	0.34 ***	0.62 ***	-					
5.	NIAS Total	0.23 *	0.73 ***	0.85 ***	0.82 ***	-				
6.	FNS Total	0.40 ***	0.52 ***	0.31 **	0.34 ***	0.49 ***	-			
7.	DEBQ Emotional Eating	0.22	0.21	−0.15	−0.05	0.00	0.25 *	-		
8.	DEBQ Restrained Eating	0.06	0.10	0.41 ***	0.30 **	0.34 **	0.25 *	0.24 *	-	
9.	DEBQ External Eating	0.02	−0.05	−0.44 ***	−0.28 *	−0.32 **	0.03	0.51 ***	−0.05	-
	Mean (SD)	21.62 (14.24)	4.61 (4.15)	3.23 (4.22)	3.73 (4.11)	11.57 (9.97)	41.96 (13.16)	2.39 (1.20)	2.76 (1.05)	3.10 (0.84)

* *p* < 0.05, ** *p* < 0.01, *** *p* < 0.001.

**Table 3:** Correlations ^1^ of sociodemographic and clinical variables with measures.

Sociodemographic and Clinical Variables	SOFAA Total	NIAS Total	FNS Total	DEBQ Emotional Eating	DEBQ Restrained Eating	DEBQ External Eating
Age	−0.11	−0.02	0.08	0.06	0.05	0.09
Gender (female)	−0.03	0.08	0.11	0.09	0.41 ***	−0.01
Education (degree)	−0.15	−0.10	0.04	0.11	0.04	0.15
Number of allergens	0.03	−0.05	0.08	−0.09	−0.06	0.10
Age at diagnosis	−0.09	0.17	−0.08	0.07	0.15	0.16
AAI prescription	0.02	−0.38 ***	0.03	−0.08	−0.30 **	0.09
Asthma	0.27 *	−0.12	0.07	0.10	−0.10	0.11
FA-related bullying	0.37 ***	0.07	0.220	0.11	0.17	0.03
FA psychological support	0.24 *	0.27 *	0.32 **	−0.11	0.06	−0.12
Current psychological issues	0.24 *	0.32 **	0.31 **	0.05	0.22	−0.03
Previous psychological issues	0.16	0.22	0.23 *	0.17	0.25 *	0.10
Previous anaphylaxis	−0.10	−0.26 *	0.09	0.21	−0.12	0.26 *
Number of reactions	0.22 *	−0.06	0.29 **	0.15	0.06	0.17
Hospital admission	0.10	−0.05	0.07	−0.07	0.04	0.01
AAI use	0.12	0.02	0.05	−0.05	0.03	−0.12
OIT	0.16	−0.04	0.08	0.02	−0.16	0.14

^1^ Pearson correlation coefficient was used for continuous and binary variables and Spearman correlation coefficient was used for ordinal variables. * *p* < 0.05, ** *p* < 0.01, *** *p* < 0.001.

**Table 4:** Multiple logistic regression model for the presence of Avoidant/Restrictive Food Intake disorder (ARFID) and Food Neophobia.

	ARFID		Food Neophobia	
Predictors	Odds Ratios	95% CI	Odds Ratios	95% CI
(Intercept)	0.20	0.00–14.09	0.05	0.00–3.77
Age	0.99	0.92–1.05	0.99	0.93–1.06
Gender	0.31	0.05–1.84	0.98	0.19–4.95
Education	2.63	0.64–12.54	1.68	0.43–7.32
Number of allergens	1.88	0.78–5.19	1.32	0.59–3.02
Age at diagnosis	1.75	0.71–4.71	1.39	0.53–3.88
AAI prescription	0.05 *	0.00–0.46	0.99	0.13–7.80
Previous anaphylaxis	4.01	0.18–136.09	1.70	0.11–29.33
Number of reactions	1.54	0.60–4.24	1.27	0.50–3.35
SOFAA total	1.02	0.97–1.08	1.14 **	1.06–1.25
DEBQ emotional eating	1.24	0.60–2.67	1.44	0.72–3.07
DEBQ restrained eating	2.49 *	1.14–6.22	2.02 ~	0.97–4.79
DEBQ external eating	0.24 **	0.07–0.63	0.43	0.13–1.23

~ *p* < 0.10, * *p* < 0.05, ** *p* < 0.01.

## Data Availability

Further data are available from the corresponding author upon reasonable request. The data are not publicly available due to privacy restrictions.
